# RAPIDIRON: Reducing Anaemia in Pregnancy in India—a 3-arm, randomized-controlled trial comparing the effectiveness of oral iron with single-dose intravenous iron in the treatment of iron deficiency anaemia in pregnant women and reducing low birth weight deliveries

**DOI:** 10.1186/s13063-021-05549-2

**Published:** 2021-09-23

**Authors:** Richard J. Derman, Shivaprasad S. Goudar, Simal Thind, Sudhir Bhandari, Zubair Aghai, Michael Auerbach, Rupsa Boelig, Umesh S. Charantimath, Rosemary Frasso, M. S. Ganachari, Kusum Lata Gaur, Michael K. Georgieff, Frances Jaeger, S Yogeshkumar, Parth Lalakia, Benjamin Leiby, Mita Majumdar, Amarjeet Mehta, Seema Mehta, Sudhir Mehta, Stephen T. Mennemeyer, Amit P. Revankar, Dharmesh Kumar Sharma, Vanessa Short, Manjunath S. Somannavar, Dennis Wallace, Hemang Shah, Manjula Singh, Sufia Askari, Mrutyunjaya B. Bellad, the RAPIDIRON Trial Group, Savitri Bendigeri, Ashwini Dadapannavar, Veerabhadra Gurlapur, Geetanjali Mungarwadi, Clavia Pereira, Neha Sharma, Tahira Parveen, Jayshree Shekhawat, Trilochan Tripathi

**Affiliations:** Thomas Jefferson University (TJU), Philadelphia, USA

**Keywords:** Anaemia, Iron deficiency anaemia, Anaemia in pregnancy, Intravenous iron, Oral iron, Low birth weight infants

## Abstract

**Background:**

Anaemia is a worldwide problem and iron deficiency is the most common cause. In pregnancy, anaemia increases the risk of adverse maternal, foetal and neonatal outcomes.

India’s anaemia rate is among the highest in the world with India’s National Family Health Survey indicating over 50% of pregnant women were affected by anaemia.

India’s Anaemia Mukt Bharat-Intensified National Iron Plus Initiative aims to reduce the prevalence of anaemia among reproductive-age women, adolescents and children by 3% per year and facilitate the achievement of a Global World Health Assembly 2025 objective to achieve a 50% reduction of anaemia among women of reproductive age.

However, preliminary results of the NFHS-5 survey completed in 2020 indicate that anaemia rates are increasing in some states and these targets are unlikely to be achieved. With oral iron being the first-line treatment for iron deficiency anaemia (IDA) in pregnancy, these results are likely to be impacted by the side effects, poor adherence to tablet ingestion and low therapeutic impact of oral iron. These reports suggest a new approach to treating IDA, specifically the importance of single-dose intravenous iron infusions, may be the key to India effectively reaching its targets for anaemia reduction.

**Methods:**

This 3-arm, randomized controlled trial is powered to report two primary outcomes. The first is to assess whether a single dose of two different intravenous formulations administered early in the second trimester of pregnancy to women with moderate IDA will result in a higher percentage of participants achieving a normal for pregnancy Hb concentration at 30–34 weeks’ gestation or just prior to delivery when compared to participants taking standard doses of oral iron. The second is a clinical outcome of low birth weight (LBW) (< 2500 g), with a hypothesis that the risk of LBW delivery will be lower in the intravenous iron arms when compared to the oral iron arm.

**Discussion:**

The RAPIDIRON trial will provide evidence to determine if a single-dose intravenous iron infusion is more effective and economically feasible in reducing IDA in pregnancy than the current standard of care.

**Trial registration:**

Clinical Trials Registry – India CTRI/2020/09/027730. Registered on 10 September 2020, http://ctri.nic.in/Clinicaltrials/showallp.php?mid1=46801&EncHid=&userName=anemia%20in%20pregnancy

## Administrative information

Note: the numbers in curly brackets in this protocol refer to SPIRIT checklist item numbers. The order of the items has been modified to group similar items (see http://www.equator-network.org/reporting-guidelines/spirit-2013-statement-defining-standard-protocol-items-for-clinical-trials/).

**Table:** 

Title {1}	RAPIDIRON: A 3-arm, randomized-controlled trial comparing the effectiveness of oral iron with single-dose intravenous iron in the treatment of iron deficiency anaemia in pregnant women and reducing low birth weight deliveries.
Trial registration {2a and 2b}.	Clinical Trials Registry – India: CTRI/2020/09/027730, registered 10/09/2020 The India Clinical Trials Registry does meet the guidelines of the WHO Trial Registration.
Protocol version {3}	Version 1.2, March 8, 2021
Funding {4}	This research is supported by a grant from the Children’s Investment Fund Foundation (CIFF) – R-1811-03347.
Author details {5a}	Thomas Jefferson University (TJU), KLE Academy of Higher Education and Research (KAHER), Jawaharlal Nehru Medical College (JNMC), Sawai Man Singh Medical College (SMSMC), Georgetown University School of Medicine, University of Minnesota, University of Alabama at Birmingham & the Children’s Investment Fund Foundation (CIFF)
Name and contact information for the trial sponsor {5b}	The sponsor for this trial is the Children’s Investment Fund Foundation (CIFF), 7 Clifford Street, London, W1S 2FT, United Kingdom
Role of sponsor {5c}	The study funder and sponsor will provide ongoing oversight of the trial by way of monthly update meetings, review of quarterly monitoring reports from the study team and providing an independent team to routinely audit the quality and safety of study practices. They will be provided data and reports for review and provide feedback but they will not have ultimate authority over study related activities.

## Introduction

### Background and rationale {6a}

Anaemia is a worldwide problem with iron deficiency anaemia (IDA) being the most common cause [1]. When occurring in pregnancy, anaemia increases the risk of adverse maternal, foetal and neonatal outcomes [2, 3]. These adverse outcomes include maternal mortality, preterm and low birth weight (LBW) deliveries, perinatal and neonatal deaths and long-term developmental sequelae in the surviving offspring [4].

Anaemia rates are among the highest in South Asia, and India’s National Family Health Survey (NFHS-4) for 2015–2016 indicated that anaemia with haemoglobin (Hb) <11.0 g/dL affected over 50% of pregnant women [5]. Anaemia during pregnancy is a major contributing factor to the nearly 60% of children 6–59 months of age classified as anaemic in the same survey with an Hb level of <11 g/dL. Optimal foetal, neonatal and childhood brain growth and development require adequate iron [6, 7]. However, women with moderate to severe anaemia during the late 2nd and early 3rd trimesters of pregnancy are often unable to make up their iron deficit. Thus, despite active transport via the placenta, insufficient iron may be transmitted to the developing foetus with consequent negative sequelae, including long-term neurodevelopmental impairment of the newborn [8].

For nearly 40 years, India’s first-line treatment for IDA in pregnancy has been oral iron; however, side effects, poor adherence to tablet ingestion and low therapeutic impact are among reasons for consideration of a new standard for treatment of IDA in pregnant women [9].

#### The Anaemia Mukt Bharat programme

The Government of India has given high priority to reducing the prevalence of anaemia in India, and several initiatives have been directed towards this objective. The latest anaemia strategy, built upon prior national programmes and supported by the Ministry of Health and Family Welfare, was presented in a 2018 publication, *Anaemia Mukt Bharat*-*Intensified National Iron Plus Initiative* [10]. The same overall strategy remains in effect, but a few changes have been made to specific intervention guidelines which can be viewed online [11].

The *Anaemia Mukt Bharat* programme encompasses a national goal of reducing prevalence of anaemia among children, adolescents and reproductive-age women at the rate of 3% a year. It also aims to facilitate the achievement of a Global World Health Assembly objective declared in 2012, to achieve a 50% reduction of anaemia among women of reproductive age by 2025 [10].

#### Existing literature

A study of Chinese pregnant women found that daily oral iron, initiated at or prior to 20 weeks’ gestation and continued until delivery, improved maternal iron parameters; however, 45% of the babies born to these women were iron deficient suggesting that oral iron supplementation may not optimally reach the developing foetus [12]. Published evidence confirms that iron deficiency in infancy is associated with an increase in cognitive and behavioural abnormalities, which may persist for decades despite later iron repletion [13, 14]. While one of the primary outcomes for this study is the risk of LBW delivery (a leading cause of under-5 mortality and an independent risk for poorer neurodevelopment), anaemia in pregnancy is associated with other adverse pregnancy outcomes such as increased rates of preterm birth and perinatal and neonatal mortality [15].

Among a cohort of 92,247 Indian and Pakistani pregnant women, in a 2019 study, 87.8% were anaemic [15]. When classified by anaemia severity, the highest percentage of pregnant women, or 49.2%, were in the moderate group (Hb 7–9.9 g/dL), and secondary analysis found that these moderately anaemic women had higher rates of poor pregnancy outcomes than those with higher Hb concentrations. For pregnant women residing in under-resourced countries and having mild anaemia (Hb 10–10.9 g/dL), research findings have not shown a consistent relationship between anaemia and poor pregnancy outcomes [4]. Thus, for this research, only pregnant women with moderate IDA will be eligible for randomization.

The Cochrane Collaboration reported a scarcity of quality trials that assessed clinical maternal and neonatal effects of iron administration to anaemic, pregnant women [16]. The Cochrane reviewers indicated the need for large, high-quality trials that assess both clinical outcomes and treatment effects. This need has not been adequately met, especially in terms of assessing the efficacy of newer treatment approaches, specifically the use of single-dose intravenous (IV) iron during pregnancy. Implementing the proposed study will address this identified but unmet need and increase the likelihood that the guidelines and recommendations of India’s respected health organizations will continue to undergo refinement. Subsequently, criteria for establishing feasibility, convenience and cost-effectiveness will be better met to enable broader and more rapid implementation and scale-up of optimal treatment approaches.

#### Intravenous iron and iron deficiency anaemia

IV iron has many advantages as a treatment of IDA. The ability to give a larger dose in a single IV injection leads to increased bioavailability of iron and more rapid correction of IDA [17–20]. This is particularly important in pregnancy to ensure iron sufficiency in the developing foetus [21–23]. In a study of women treated with IV iron, none of the newborns were diagnosed with iron deficiency anaemia [24]. Furthermore, this method reduces the risk of poor adherence to treatment so often seen with oral iron. Study findings also indicate that IV iron is safe (with no serious adverse events reported), less toxic and more effective than oral iron in the treatment of IDA in pregnant women [25, 26].

This study will be a 3-arm, randomized-controlled trial comparing the effectiveness of two IV iron formulations: ferric carboxymaltose and ferric derisomaltose (formerly known as iron isomaltoside), with oral iron in the treatment of IDA in pregnant women and in reducing low birth weight deliveries [27–29]. Two IV iron formulations, approved for marketing in India, will be used with the aim of demonstrating that pregnant women, randomly assigned to either of the formulations, will have a higher rate of conversion to a non-anaemic state when compared to the oral iron comparator arm.

#### Summary

This research aims to assess the efficacy of a single dose of IV iron, administered early in the second trimester of pregnancy for the treatment of moderate IDA, in achieving a greater percentage of women with a normal for pregnancy Hb concentration of >11 g/dL by delivery, when compared to a control arm provided with an oral iron regimen that is the primary and current standard of care in India.

Published data suggest that IV iron is a safe and more effective treatment of IDA in pregnancy than is oral iron. This newer approach to the treatment of IDA must be ideally evaluated through a randomized control trial in a population that continues to be greatly affected by this problem and has a critical need to employ more effective management strategies.

### Objectives {7}

#### Study hypotheses

This trial incorporates the following study hypotheses:

##### Primary hypothesis #1

Singleton pregnant participants with moderate iron deficiency anaemia who are randomly assigned to an IV iron arm and receive, early in the second trimester of pregnancy, a single dose of IV iron for treatment of anaemia and the currently recommended daily dose of folic acid will have a higher conversion rate to non-anaemic status (or Hb >11 g/dL) in the last trimester of pregnancy at either a 30–34 week antenatal visit or prior to delivery than pregnant women assigned to an oral iron arm and provided a standard dose of iron and folic acid tablets for anaemia treatment.

##### Primary hypothesis #2

The pregnant participants assigned to an IV iron treatment group will have a lower risk of LBW deliveries (a 20% relative reduction) compared to pregnant women in the oral iron group.

##### Secondary hypothesis

To determine if administration of either of the two IV iron formulations used in the study will have a more favourable impact on other maternal and neonatal outcomes than among women provided daily oral iron. This will be assessed through reported differences between study arms at specified timepoints for the secondary outcomes listed in section {12}.

### Trial design {8}

This study is a 3-arm randomized-controlled trial with two primary outcomes of interest. The research is designed to assess if a single dose of an IV iron formulation (ferric carboxymaltose in intervention arm 1 or ferric derisomaltose (formerly known as iron isomaltoside) in intervention arm 2), provided for treatment of moderate IDA in pregnant women, will result in a greater percentage of pregnant participants achieving a normal for pregnancy Hb concentration of >11 g/dL by the third trimester, when compared to the comparator arm (arm 3) given oral iron. Moderate IDA has been defined employing the WHO definition for all cause anaemia severity, Hb < 7 g/dL (severe); 7–9.9 g/dL (moderate); 10–10.9 g/dL (mild); and >11 g/dL (non-anaemic or normal) [30]. Those eligible to receive iron treatment must also show laboratory values of serum transferrin saturation (TSAT) < 20% and/or ferritin < 30 ng/mL.

The second primary outcome is that of low birth weight (LBW) (< 2500 g), one of several adverse pregnancy outcomes associated with IDA. The hypothesis for reporting on this clinical outcome is that the risk of LBW delivery for participants randomized to the IV iron arms will be significantly lower when compared to the risk of LBW delivery for participants randomly assigned to the oral iron arm.

Approximately 4320 pregnant women who meet eligibility criteria and have Hb concentrations of 7 to 9.9 g/dL with confirmed IDA will be randomized 1:1:1 to one of two IV iron intervention arms or to the oral iron arm.

## Methods: participants, interventions and outcomes

### Study setting {9}

The study will be carried out in India with participants recruited during an antenatal care visit made to participating community health and primary health centres (CHCs and PHCs) in the states of Karnataka and Rajasthan.

### Eligibility criteria {10}

Inclusion criteria for screening and consent are as follows: (1) pregnant women between 18 and 40 years of age and capable of giving informed consent; (2) Hb concentration of 7–10.4 g/dL (as per the available point of care laboratory report); (3) expressed intent and expectations for remaining in the designated research area during the pregnancy and delivering at a facility in or near the research area as well as remaining in the area to enable study participation and data collection consistent with the research protocol; and (4) expressed willingness that specifically includes agreement to randomization to the standard care study arm (of oral iron) or to one of the two arms involving treatment with single-dose IV iron.

Additional inclusion criteria for randomization and continued study participation include (1) presence of a live singleton, intrauterine foetus and dating ultrasound that indicates a pregnancy that, at randomization, would be between the beginning of week 14 and prior to 17 weeks 0 days, and (2) iron deficiency anaemia defined for this study as moderate anaemia with Hb concentration level between 7 and 9.9 g/dL and serum transferrin saturation (TSAT) < 20% and/or serum ferritin < 30 ng/mL.

Exclusion criteria include (1) foetal anomaly if detectable when an initial ultrasound is done to date the pregnancy (subsequent discovery of a foetal anomaly is not viewed as an exclusion); (2) a history of cardiovascular disease, hemoglobinopathy or other disease or condition considered a contraindication for treatment, including conditions recommended for exclusion by the manufacturers of oral or IV iron to be used in the study; and (3) any condition that, in the opinion of the consenting healthcare provider, warrants study exclusion.

### Who will take informed consent? {26a}

Women will be screened for their eligibility to participate in the study at their first antenatal visit at participating CHCs and PHCs. If no exclusionary criteria are known to apply, pregnant women will be educated about the study, have all their questions answered and be given the opportunity to consent to participate. Consent will be obtained by a trained healthcare provider or research staff member at the CHC or PHC.

Following review of the consent and medication procedures, the woman will be asked to confirm and document her willingness to participate in the study by signing the consent form. If the participant is unable to sign, her thumbprint will indicate written approval. Both the staff member and the study participant will retain signed copies of the form. If a prospective participant is not prepared to consent she will be given the option to take the form home to discuss participation with her family before signing.

All staff responsible for obtaining consent will be trained and certified in the protection of human subjects and study-specific consent procedures. Each site will be provided with a model informed consent form, developed by the research team.

### Additional consent provisions for collection and use of participant data and biological specimens {26b}

When obtaining informed consent, participants will be informed of which biological specimens will need to be collected, at what timepoint as well as the reason for such studies. They will also be informed that their data will be de-identified and stored in a secure database and that any data released will not be identifiable.

The consent procedure includes a condition wherein participants are specifically asked to consent to the use of data collected as part of the study or information about outcomes under study for unrestricted scientific purpose(s) only. They are also given the option to consent to being contacted in the future regarding participation in other related research studies. Participants will be told they can withdraw consent at any time during the trial.

### Interventions

#### Explanation for the choice of comparators {6b}

This 3-arm study will incorporate two IV iron formulations, ferric carboxymaltose in arm 1 and ferric derisomaltose in arm 2, and a comparator arm of a standard dose of oral iron in arm 3. Two IV iron formulations are being compared with the aim of demonstrating effectiveness of either in the treatment of IDA when compared to oral iron.

Both IV iron formulations selected for use in this study are approved and available commercially in India, and both are proposed for the following reasons: (1) they allow single-dose infusions of up to 1 g of iron, (2) they have a proven track record of efficacy and availability in many countries of the world, (3) they are associated with very low rates of adverse events, (4) high-quality studies show no difference in severe side effects among available IV iron formulations and (5) there is greater probability that inclusion of more than one single-dose formulation will be instrumental in driving down market prices and perhaps lead to public sector pricing and greater utilization.

Oral iron will be used as the comparator arm as this is currently the first-line treatment for mild and moderate IDA in pregnancy.

#### Intervention description {11a}

Ferric carboxymaltose (Revofer Lupin) will be sourced in 500-mg vials. Ferric derisomaltose (Jilazo Lupin) will also be sourced in 500-mg vials.

Pregnant women randomly assigned to an IV iron arm at the third study visit will receive their single-dose IV infusion at a participating CHC. Prior to beginning the infusion, the pregnant woman will be counselled about the infusion procedure and the risks and benefits before confirming her willingness to proceed.

If the participant weighs more than 50 kg, they will receive the assigned single dose formulation of 1000 mg of iron; but women under 50 kg will receive a lower dose of iron as determined by a formula that has been recommended by both manufacturers of 20 mg iron/kg body weight.

Oral iron will be given to arm 3 in the form of 60mg Ferrous Sulphate tablets given to anaemic women twice daily.

In all three study arms, participants will receive 180 tablets of folic acid (500 mcg) once daily and one tablet of albendazole (400 mg) after randomization in the second trimester of pregnancy.

#### Criteria for discontinuing or modifying allocated interventions {11b}

Interventions will be discontinued at any time under the following circumstances: (1) if the participant withdraws consent; (2) if the investigator determines the intervention is a threat to the participant’s health, safety and/or wellbeing; or (3) if the participant experiences adverse effects requiring the intervention be discontinued.

#### Strategies to improve adherence to interventions {11c}

Accredited Social Health Activists (ASHAs) who live within the communities of recruiting CHCs and PHCs will support the study team (with authorization of the supervising health officials) and will be available to accompany potential participants to facilities for screening, dating of the pregnancy (by ultrasound), for randomization followed by treatment of anaemia, antenatal monitoring and delivery, and the 42-day post-delivery visit. ASHAs who have received education on IDA can be an additional source of information about the study.

Participants in the oral iron arm will be given clear instructions about how to take the tablets and will be asked at subsequent visits if they are taking their medication and if not, the reasons for this. Answers will be recorded on appropriate case report forms (CRF).

During the consent process, potential participants will be informed about the randomization process and procedures if they are found eligible for randomization—that is, they will either be provided oral iron and other components of standard care or be given a single IV iron infusion. Participants will be counselled on the risks and benefits of treatment relevant to all study arms during the consent process.

Once randomized, and regardless of treatment arm, participants will be encouraged to follow the treatment plan for their study arm and attend the study visits as instructed. Visits follow a schedule of standard antenatal care that will also provide an opportunity to monitor participant progress and encourage adherence to the treatment plan.

Noncompliance with the plan for iron supplementation may be less for women randomly assigned to an IV iron intervention as supplementation during pregnancy would typically be complete after receipt of a single IV iron infusion unless referral, assessment and additional treatment is required.

#### Relevant concomitant care permitted or prohibited during the trial {11d}

Unscheduled healthcare visits may occur for an enrolled, randomized study participant. If this occurs, information about this visit will be collected on a specific unscheduled healthcare visit CRF.

Oral iron should not be taken by a pregnant woman that receives an IV iron infusion in accordance with the manufacturer guidelines. If a participant takes oral iron having received an IV iron infusion, a protocol deviation would be noted.

Any use of oral iron, outside of that used by participants in the oral iron arm of the study, is considered an exclusion criterion.

The antenatal care provider seeing an enrolled and randomized study participant will make decisions about care that may be required because of other (non-anaemic) pregnancy complications or other health-related issues. It is expected that the provider will offer standard of care to the pregnant woman in treating non-anaemia issues or other non-pregnancy related health concerns that may arise.

#### Provisions for post-trial care {30}

The last scheduled study visit occurs at approximately 42 days postpartum. It would be expected that healthcare providers will deliver standard of care and specify the need for follow-up care for both the mother and baby as deemed necessary.

### Outcomes {12}

#### Primary outcomes

The first of two primary outcomes is to evaluate whether a single dose of IV iron (ferric carboxymaltose in intervention arm 1 and ferric derisomaltose in intervention arm 2, administered early in the second trimester of pregnancy for treatment of moderate IDA, will result in a greater percentage of pregnant participants in the IV iron arms achieving a normal for pregnancy Hb concentration of >11 g/dL in the third trimester of pregnancy, confirmed at either a 30–34-week antenatal visit or prior to delivery, when compared to those in the oral iron comparator arm (arm 3).

The second primary outcome is to evaluate the incidence of low birth weight (< 2500 g), one of several adverse pregnancy outcomes reported to be associated with IDA. The hypothesis for this clinical outcome is that the risk of LBW delivery for participants randomized to the IV iron arms will be 20% lower when compared to the risk of LBW delivery for participants randomly assigned to the oral iron arm.

#### Secondary outcomes

Among the secondary outcomes to be recorded are changes in Hb concentration, variation in participant iron indices at specified timepoints (TSAT and ferritin, changes in cord blood Hb and other iron indices of cord blood (Fe/TIBC (TSAT) and ferritin), weight gain of participants by trimester of pregnancy, mode of delivery/C-section, antepartum and severe postpartum haemorrhage, hypertensive disorders, maternal or neonatal infections including documented COVID-19, maternal and neonatal mortality, preterm and small for gestational age births, pregnancy loss and stillbirths, birth weight of live born babies, newborn length, the need for neonatal resuscitation, neonatal admissions to an intensive care unit, time from delivery to cord clamping, unanticipated or extended hospitalizations, breastfeeding practices and maternal well-being (quality of life).

In consideration of current *Anaemia Mukt Bharat* interventional guidelines for anaemia treatment in pregnant women [11], two additional secondary outcomes will be assessed: (1) participant need for “rescue therapy” or measures implemented for management of severe anaemia after a drop in Hb concentration to <7 g/dL at any time after treatment is initiated and (2) referral of a participant, regardless of study arm, to a higher level of care (CHC, Taluka hospital, or District Hospital/Tertiary Care Centre) for further investigation of the cause of anaemia due to <1 g/dL improvement in Hb based upon analysis of blood collected at 26–30 weeks of pregnancy.

### Participant timeline {13}

The projected timeline for this study is approximately 42 months and will consist of a 7-month preparatory phase, 28 months for recruitment, treatment and follow-up, as well as a final 5-month phase for data cleaning and analysis. This is summarized in Table 1.

**Table 1:**
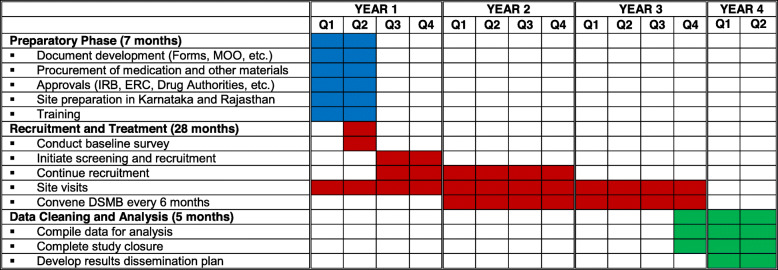
Study timeline

#### Sample size {14}

The sample size for *RAPIDIRON* is primarily driven by the comparison of LBW risk. The true risk of LBW is assumed to be 25% in the oral iron arm based upon the LBW estimate of 21% found in a similar population with a Hb level at delivery of 7–9.9 g/dL but with fewer rural sites [15]. Parks et al. estimate used data from geographic clusters participating in the *Eunice Kennedy Shriver* National Institute of Child Health and Human Development (NICHD) Global Network’s Maternal Newborn Health Registry for Jawaharlal Nehru Medical College’s (JNMC) as well as data from the same registry for clusters located near Nagpur, India, and in Pakistan.

Assuming a true LBW risk of 25% in the oral iron group, a final sample of 1332 per group would give 80% power to detect a 20% relative reduction (relative risk of 0.8; 20% LBW risk in each of the IV iron groups) at the alpha=0.0245 level. One interim analysis when 1/3 of the information on LBW is available is planned. With *p*-value boundaries of 0.000026 at the interim analysis and 0.02449 at the final analysis, based on 10,000 simulations, with evaluable data on 1332 participants per arm (444 per arm at the interim analysis), and assuming both IV iron arms have true risk of LBW of 20%, the study design provides a 1.6% probability of stopping early for efficacy, approximately 80% probability of concluding an a priori-selected IV arm has lower LBW risk than the oral arm, and greater than 90% probability of concluding at least one of the IV arms is better than the oral iron arm. Approximately 4320 pregnant women will need to be randomized 1:1:1 to each of the three study arms assuming that 7.5% or less of participants have missing birth weight data (due to stillbirth and other reasons). Each of the two research sites (based in either Karnataka or Rajasthan) will enrol and randomize no more than 60% of all study participants.

With 1332 study participants per group, the study provides greater than 95% power to detect differences in risk of non-anaemia of at least 10% at the alpha=0.0005 level; as such the study is well powered for this outcome assuming that the true risk approaches the anticipated difference of 30% based on similar studies.

#### Recruitment {15}

Approximately 16,000 pregnant women will be screened for low Hb at their first antenatal visit at participating CHCs and PHCs. This sample size should achieve an adequate number of pregnant women who are subsequently determined eligible to be randomized equally to each of the three study arms. Recruitment and randomization of participants will continue until study data provide confidence that a minimum of 1332 randomized participants in each arm have evaluable primary outcome data.

### Assignment of interventions: allocation

#### Sequence generation {16a}

Randomization will be stratified by enrolment site, with the two Indian research sites of Belagavi, Karnataka and Jaipur, Rajasthan comprising the two randomization strata. Randomization sequences will be generated for each stratum using a computer-generated algorithm based upon a randomly permuted block design with randomly varied block sizes as specified in a detailed randomization plan to which all study investigators, other than the coordinating centre randomization statistician, are masked.

#### Concealment mechanism {16b}

The block sizes will be known only to personnel responsible for the randomization algorithm. Randomization assignments will be placed in sequentially numbered opaque envelopes by a designated pharmacist, grouped by sets of 12, and stored at the central research coordinating office of each research site. Envelopes will be distributed monthly to each CHC/PHC in sets of 12 (e.g. set 1 [envelopes 1–12] to PHC 1, set 2 [envelopes 13–24] to PHC 2, sets 3 and 4 [envelopes 25–48] to PHC 3, etc.) based on projected enrolment at the CHC/PHC for that month.

#### Implementation {16c}

Randomization assignments will be generated by the statistical team at Thomas Jefferson University (TJU). The sealed envelopes containing iron tablets/IV iron will be available at the CHCs and PHCs. Accordingly, the Medical Officer will randomize the participant. If the participant is allocated to IV iron arm, then the Medical Officer will calculate the dose and write it on the randomization slip. The same randomization sequence numbered envelops with iron tablets/IV iron (either ferric derisomaltose/ferric carboxymaltose) will be available at the CHCs. The pharmacist at the CHC will match the randomization number on the envelope issued at the CHC or PHC. Upon confirmation of the number and opening of the envelope which designates either ferric derisomaltose/ferric carboxymaltose, the pharmacist will prepare the infusion (dose as per the instructions written by a Medical Officer at the CHC or PHC during randomization). The dose is double-checked  based on the patient’s weight with subsequent dispensing and provided to a staff nurse who will administer the IV iron to the participant under the supervision of a CHC Medical Officer.

### Assignment of interventions: blinding

#### Who will be blinded {17a}

The investigators at JNMC in Belagavi and SMSMC in Jaipur will identify a specifically designated pharmacist who will be assigned to the research team in their respective site. They will assure compliance with the specified randomization process and receipt of the correct iron supplementation modality for each participant. Oral iron use will not be masked. However, blinding will occur for the two IV iron arms since IV iron participants will know only that they have been randomly assigned to receive an IV iron infusion without knowing the specific arm (formulation) that will be provided. Further, neither the point of care provider nor the infusion team will know which IV iron formulation was assigned. However, each pharmacist responsible for preparing the IV Iron infusions for the trial will not be blinded. When a participant arrives for an infusion, the pharmacist will collect an infusion order provided to the participant and prepare the appropriate IV formulation for infusion based on the randomization ID list. The IV medication will ONLY be known to the pharmacist, with all other clinical staff blinded as to which of the two IV formulations is being used.

#### Procedure for unblinding if needed {17b}

The participant consent form clearly indicates that if there is a requirement to release study information regarding participant treatment associated with receive emergency care if needed, an authorized staff person has permission to gain access to a protected participant record. All episodes of unblinding will be shared with members of the Data and Safety Monitoring Board (DSMB).

### Data collection and management

#### Plans for assessment and collection of outcomes {18a}

A baseline survey will be conducted in at the CHCs and PHCs in both Belagavi and Jaipur. This survey consists of 3 questionnaires, one to be distributed to each of the following 3 groups: (1) pregnant women between 24 and 34 weeks of gestation (thus not eligible for inclusion in this trial), to explore the current practices about antenatal care, compliance with anti-helminthic, iron and folic acid, delivery details and out of pocket expenditure; (2) healthcare providers to better understand supply of resources to CHCs/PHCs and the management of anaemia at these centres; and (3) administrators to gain an understanding of the supply chain of medicines provided to CHCs/PHCs especially those medications utilized in the RAPIDIRON trial.

A summary of the study procedures including outcomes to be collected at specific study related visit can be found in Table 2.

**Table 2:**
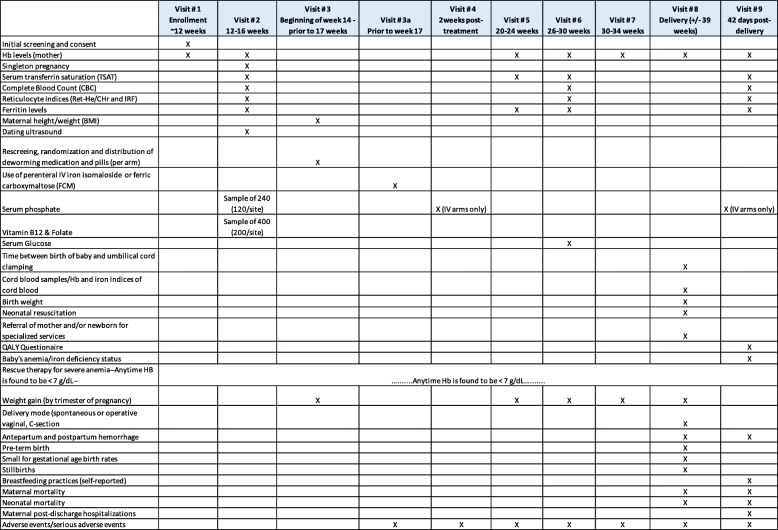
Timing of study procedures

All personnel will undergo training to familiarize themselves with the trial protocol. There will also be appropriate training in research ethics and employment of Good Clinical Practice (GCP) guidelines.

Study staff involved in administering and monitoring the IV iron infusions will receiving procedure-specific training and access to the study Manual of Operations (MOO) for reference as needed.

Trained ultra-sonographers will be selected to perform ultrasound examinations. They will undergo study-specific training before starting to scan participants for eligibility based upon gestational age. Detailed information on ultrasound procedures and standardization will be available to staff in the study MOO. The American College of Obstetricians and Gynaecologists (ACOG) provides guidance on due date estimation using ultrasonography when an accurate Last Menstrual Period (LMP) is unavailable. This information can be utilized by study team as needed and is described in the study MOO.

Equipment used in the study will be standardized throughout the study sites including weighing machines, ultrasound equipment and laboratory equipment/assays.

#### Plans to promote participant retention and complete follow-up {18b}

The role of the ASHAs (as described in the section “Strategies to improve adherence to interventions {11c}”) is to act as facilitators to promote participant clinic attendance and retention of study participants.

Regardless of whether participants discontinue treatment or deviate from the intervention protocol, all information from participants will be collected through the end of the trial, unless the participant withdraws such consent.

#### Data management {19}

JNMC is responsible for the development of the independent Data Management System (DMS) and utilization of the database system for the *RAPIDIRON Trial.* Data will be collected primarily through the use of paper-based forms. JNMC has successfully used the Research Electronic Data Capture (REDCap) system for numerous research projects. This system is a secure web application for building and managing online surveys and databases, and it offers a secure, privacy compliant web-based electronic data capture and maintenance system. The data are stored in a centralized system. REDCap software requires each user to have their own account, and user privileges are applied to ensure that users have access only to data and information they need and are authorized to have. REDCap can be accessed from multiple sites from any computer or mobile device, including PCs, Macs, tablets and mobile phones.

Data will be collected at the CHCs/PHCs and transferred to regional data centres for data entry and form storage and will be entered into computers using the data management system developed by the research team at JNMC. The data will then be transferred to TJU utilizing REDCap. The DMS will involve a REDCap server host and include project setup and form building with validations and ranges.

Data quality validation with inbuilt quality checks will be programmed into REDCap with external quality control. Routine reports will be developed to include missing forms, expected forms and participant follow-up schedules.

The data entry staff at each research site is responsible for entering data from all completed paper forms into the DMS. If data corrections are needed, study staff will enter the corrections into the DMS, which will create an automatic audit trail. The DMS retains built-in quality checks to verify the accuracy and quality of the data entered.

#### Confidentiality {27}

At the time of enrolment, each participant will be given a unique screening ID number. All subsequent data collection will be linked only to the ID number rather than to a participant name.

In addition, the DMS is password-protected to ensure that only trained, authorized personnel have access to the data. The consent forms and locator forms linking personal information to ID will be kept in securely locked filing cabinets at each research site with only authorized staff having access. If unauthorized use of participant information is discovered, the onsite staff must notify the senior research team and Principal Investigator (PI) along with plans to correct the problem and prevent further violations.

#### Plans for collection, laboratory evaluation and storage of biological specimens for genetic or molecular analysis in this trial/future use {33}

Blood samples will be collected at study-related visits as detailed in Table 2. Samples will be collected at the locations detailed in Table 3. As two laboratories will be utilized, one at each study site, each employing daily controls, these processes will provide additional validation of results.

**Table 3:** Blood sample collection sites

Visit number	Collection site
Visit #2: (12–16 weeks’ gestation)	Respective hospitals in Belagavi and Jaipur Belagavi: KLES Dr. Prabhakar Kore Charitable Hospital and Medical Research Centre Jaipur: SMS Medical College and Hospital
Visit #4: 2 weeks post-treatment	CHC/PHC
Visit #5: 20–24 weeks gestation	CHC/PHC
Visit #6: 26–30 weeks’ gestation	CHC/CHC
Visit #7: 30–34 weeks’ gestation	CHC/PHC
Visit #8: At delivery	Participant’s Delivery Location
Visit #9: 42 days post-delivery	CHC/PHC

A mobile research team will be utilized in this trial to ensure the necessary assessments and sample collections are performed as planned. They will be responsible for carrying out study related procedures including: assisting Medical Officers in screening and enrolment, arranging transport to a central facility for eligibility assessment, obtaining study-specific blood draws at scheduled visits, providing appropriate newborn weight and height assessment within 72 h of delivery and maintaining study logs. They will be contacted by a participant’s CHC/PHC/birthing facility/ASHA/relative or the participant whenever a delivery is anticipated or their presence is required.

Immediately after collection, the samples will be stored upright at 2 to 8 °C. Samples should be transported from the CHCs/PHCs to the central lab within 6 h of collection. Those specimens ready for transport will be stored in an insulated chiller box containing ice packs and a thermometer to record the transportation temperature. Once specimens arrive at the laboratory, the laboratory staff who receives the samples must complete the Laboratory Requisition Form (LRF). The specimens will then be subjected to processing at the respective site laboratories: KLE Hi-Tech Clinical Lab, Belagavi and SMS MC Clinical Lab Jaipur.

The following procedures will be carried out to ensure quality control of sample analysis throughout the trial: (1) the Lyphochek Anaemia Control (BIORAD). Analytes: Ferritin, Folate, Iron (TIBC/UIBC), Vitamin B12. Such controls will be run each time study samples are to be analysed; (2) an internal QC process has been established for study samples; (3) both laboratories will participate in protocol-specific EQA testing; and (4) proficiency programme provider: BioRad for Biochemical assays and All India Institute of Medical Sciences (AIMS)/Christian Medical College (CMC) Vellore for haematology parameters.

### Statistical methods

#### Statistical methods for primary and secondary outcomes {20a}

The primary analysis for anaemia will compare each IV iron arm to the oral iron arm with respect to the percentage of participants who achieve Hb of >11 g/dL at either a 30–34-week antenatal visit or prior to delivery using a Cochran-Mantel-Haenszel (CMH) chi-square test stratified by enrolment site at the 0.0005 level. The CMH-adjusted risk ratio and associated 99.95% confidence interval will be calculated for both comparisons. No interim efficacy analysis is planned for the anaemia outcome.

Each IV arm will be compared to the oral iron arm with respect to risk of LBW using a CMH chi-square test stratified by enrolment site at the 0.000026 level when data are available on at least 444 participants per group (1/3 of expected information). The study can be stopped for efficacy if either IV arm shows significantly lower risk of LBW at the interim analysis. Assuming that the study is not stopped early, the final tests will be performed at the 0.02449 level. Stopping boundaries were calculated using a Lan-DeMets spending-function approach with O’Brien-Fleming bounds. The CMH-adjusted risk ratio and associated 97.55% confidence interval will similarly be calculated for both comparisons. If the study is prematurely stopped early based on the results of LBW data, analysis of the anaemia endpoint will be performed at the 0.025 level.

The primary analysis will be performed using the intent to treat (ITT) cohort. Missing anaemia and LBW data will be imputed using multiple imputation. The exact method of imputation will be specified in the statistical analysis plan prior to analysis. Fifty data sets will be imputed and the Wilson-Hilferty transformation will be applied to the CMH statistics fit to each data set [31, 32]. Results will be pooled using the method of Rubin to calculate the overall test of significance [33]. The log (relative risk) estimates of each imputed data set will similarly be combined.

Analysis of categorical secondary outcomes with respect to treatment arm differences will use generalized linear models to estimate relative risks and associated confidence intervals. Analysis of continuous, longitudinally measured, secondary outcomes with respect to treatment arm differences will use mixed effects linear regression, accounting for correlation among repeated measurements over time and adjusting for site as a random effect. Mean differences between randomization groups will be estimated from the model results at each measurement time along with appropriate confidence intervals.

Association of Hb levels at delivery with LBW delivery will be evaluated using logistic regression. For this analysis, Hb levels will be categorized using the WHO definitions for severity: < 7 g/dL (severe), 7–9.9 g/dL (moderate), 10–10.9 g/dL (mild) and >11 g/dL (non-anaemic or normal). If one or both of the IV iron formulations are demonstrated to be effective in transitioning women to non-anaemic status (Hb >11g/dL) in the third trimester of pregnancy at either or both of two timepoints prior to delivery, or if one or both IV iron formulations are effective in reducing the risk of LBW deliveries, a series of secondary exploratory analyses will be performed to identify baseline factors that are predictive of treatment success. The objective of these analyses will be to support future implementation of the treatment regimen in broad populations by identifying subpopulations most likely to benefit from the treatment regimen. Separate logistic regression models will be constructed for the anaemia and LBW outcomes, with predictors to include Hb levels at the time of randomization as well as TSAT and ferritin levels and other baseline characteristics identified in the Statistical Analysis Plan.

#### Interim analyses {21b}

An interim analysis for efficacy for the LBW outcome will be performed when data are available on at least 444 participants per treatment arm (1/3 of expected information). Using stopping boundaries from a Lan-DeMets spending function with O’Brien-Fleming bounds, we set *α*_1_=0.000026 so that the study could be stopped if the comparison of either IV arm to the oral arm had *p*<0.000026. Assuming the observed risk of LBW is 25% in the oral arm, this would occur if the observed risk of LBW in a particular iron arm were 13.7% or less. To maintain an overall type-I error rate of 0.0245 for each IV vs. oral comparison, if the null hypothesis is not rejected at the interim analysis, the final comparisons for LBW will be completed at the 0.02449 level.

Assuming the study is not stopped for efficacy, one formal interim analysis for futility will be completed based on a data snapshot taken when the anaemia outcome is available for 666 participants per group (1/2 of expected information). The interim analysis for futility will involve the conditional power associated with two hypothesis tests: the two-degree of freedom CMH test of differences in prevalence of anaemia prior to delivery across the three treatment arms and the two-degree of freedom test of differences in risk of LBW across the three treatment arms. If analyses for both primary efficacy outcomes show a conditional power of less than 0.2 based on the observed data at the time of the interim analysis and the assumed effect for the study design, the DSMB may consider recommending that the study be stopped.

The analysis for safety will have two components. First, point and 97.5% confidence interval estimates of the risk of mortality and incidence of at least one SAE will be developed for each of the three treatment arms. If the interval estimates provide evidence that either of the active IV iron treatment arms has significantly higher risk of either mortality or adjudicated SAE incidence than the active comparator arm (oral iron), the DSMB will consider recommending stopping enrolment for that active IV iron treatment arm. In making the recommendation, the DSMB can consider the overall risk profile of the treatment arms and any evidence of efficacy that has been accumulated at the time of the interim analysis.

#### Methods for additional analyses (e.g. subgroup analyses) {20b}

IV iron arms will be descriptively compared with respect to the proportion of LBW births and proportion of non-anaemic patients by estimating the CMH-adjusted risk ratio and associated 95% confidence interval. An additional analysis comparing each IV iron arm to oral iron will be performed on the per protocol cohort.

An exploratory Hb-based subgroup analysis will be performed to estimate the effect of treatment on conversion to non-anaemia status and the risk of LBW solely by level of Hb measured prior to randomization (between the beginning of week 14 and 17 weeks 0 days of pregnancy). Hb-based subgroups will be defined based on clinical considerations but may be collapsed depending on the sample sizes within each group.

#### Methods in analysis to handle protocol non-adherence and any statistical methods to handle missing data {20c}

The ITT cohort includes all randomized study participants using treatment group assignments as randomized. Missing anaemia and LBW data will be imputed using multiple imputation as described above. The exact method of imputation will be specified in the statistical analysis plan prior to analysis. Secondary outcomes will not be imputed.

The per-protocol cohort includes all study participants randomized to IV who receive the complete, recommended dosage of the randomly assigned IV iron formulation between the beginning of the 14th week of pregnancy and prior to the beginning of the 17th week and all study participants assigned to oral iron therapy who take equal to or greater than 80% of prescribed pills. Use of any non-study-provided iron preparation post-randomization will represent an exclusion for the purpose of the per protocol analysis. Additionally, a participant assigned to an IV iron arm that receives only a portion of the recommended single infusion dosage for the randomly-assigned IV iron formulation will be excluded.

#### Plans to give access to the full protocol, participant-level data and statistical code {31c}

Data sharing will be granted in accordance with the details registered with the Clinical Trial Registry of India (CTRI). Individual participant data that underlie the results reported in specific publications arising out of the trial, after de-identification (text, tables, figures and appendices) will be shared. Additionally, the study protocol, statistical analysis plan and informed consent forms will be shared.

These files will be viewable by researchers whose proposed use of the data has been approved by the Trial Steering Committee. The data will be available for analyses necessary to achieve aims in the approved proposal. Data will be available via communication with representatives of the Trial Steering Committee by email: Dr Richard Derman (Richard.Derman@jefferson.edu) and Dr M B Bellad (mbbellad@hotmail.com). Data will be available beginning 3 months and ending 5 years following article publication.

### Oversight and monitoring

#### Composition of the coordinating centre and trial steering committee {5d}

The Trial Steering Committee consists of the study PI, Dr. Richard Derman; co-investigators from the Belagavi site, Dr. Shivaprasad Goudar and Dr. Mrutyunjaya Bellad; and co-investigators from the Rajasthan site, Dr. Sudhir Mehta and Dr. Kusum Gaur. Each of the 3 collaborative research groups involved in this trial at Belagavi, Jaipur and TJU will have established a senior leadership team with team members meeting on a weekly basis. This specified Steering Committee will be responsible for overall oversight and governance of the trial with the final responsibility remaining with the PI.

The trial coordinating centre will reside at JNMC and having responsibility for coordinating daily operations for both the Belagavi and Jaipur sites. This includes ongoing training and management of study site staff.

The study Technical Advisory Group (TAG) will convene on a biannual basis to provide technical assistance and oversight.

#### Composition of the data monitoring committee, its role and reporting structure {21a}

An independent DSMB will be comprised of five individuals: a chair and four supporting members. All members of the DSMB will be free of conflicts of interest to enable performance of their duties in an unbiased manner.

The duties to be performed by the DSMB include reviewing serious adverse events (SAEs), and adverse events (AEs) and unanticipated problems posing risks to study participants or their foetuses/offspring; assuring that the research is conducted in a manner to minimize risk and promote safety; assessing data quality, participant recruitment, accrual and retention; and preparing reports that outline DSMB recommendations and providing them to the research team. The DSMB will meet approximately every 6 months until all study participants have been followed and data collected on outcomes through 42 days post-delivery. Further information on the DSMB can be found in the study protocol and MOO.

A designated Chief Quality and Safety Officer will also serve as the senior biostatistical consultant for the *RAPIDIRON Trial.* This individual will work with the Data Management System (DMS) at JNMC and the senior statistician at TJU to monitor the ongoing quality of study operations and safety of study participants. He will also serve as the primary interface between the study investigators and the DSMB.

Given the importance of distinguishing serious adverse drug reactions specifically related to intravenous iron infusions from non-serious reactions that commonly occur with the use of any intravenous medication, two experts familiar with the use of IV iron have been designated as adjudicators.

The study teams from both Belagavi and Jaipur will report possible SAEs in a timely manner to the adjudicators. Two adjudicators will conduct rapid reviews and provide case by case validation of all SAEs and comment on the clinical management of each of these cases. These reports will be submitted to the study PI and site PIs, who will share the information with the DSMB in a timely manner.

#### Adverse event reporting and harms {22}

An adverse event (AE) is defined as any untoward medical occurrence in a study participant. Any AE that occurs during the course of the trial and requires treatment should be reported and the AE CRF completed.

The RAPIDIRON trial is recruiting pregnant women as participants. It is expected that these women will experience a range of obstetric-related symptoms during pregnancy, labour and the postpartum period. These should not be recorded as AEs unless the event is of unusual severity, intensity, duration or frequency.

When uncertainty exists as to whether a condition is to be reported as an AE or SAE such cases should be reported to and monitored by the adjudicators.

A serious adverse event (SAE) involves a randomized participant that initiated treatment OR the foetus or newborn of such a participant. To qualify as an SAE, at least one of the following criteria must apply to the event: (1) results in a maternal or neonatal death, or a foetal death greater than 20 weeks’ gestation; (2) is life-threatening; (3) requires an unanticipated hospitalization or prolongs an existing hospitalization; (4) results in persistent or significant disability or incapacity; or (5) represents other serious or unexpected adverse events that a study investigator feels should be reported, including need for resuscitation.

Study drug reactions will be reported as an SAE if they meet an SAE criterion (above) and will be documented as an SAE that is related to the use of the study drug only when confirmed by adjudication.

SAEs will be reported to, and monitored by, the DSMB.

At each scheduled DSMB meeting after study recruitment, SAE/AE data and a report summarizing recruitment and participant status will be prepared for review and discussion. Following its deliberations, the DSMB will issue any recommendations it may have and specify that the study should continue based upon absence of significance safety concerns.

#### Frequency and plans for auditing trial conduct {23}

The study PI and statisticians will meet monthly to review the data and evaluate its quality and safety. They will provide a monitoring report with deidentified data for the study funder on a quarterly basis. The data will also be reviewed by an independent DSMB who will meet every 6 months throughout the trial.

The study funder (CIFF) will employ an independent clinical research organization (CRO) to routinely audit the study and monitor the quality and safety of study practices.

#### Plans for communicating important protocol amendments to relevant parties (e.g. trial participants, ethical committees) {25}

The senior research team will meet weekly to review all concerning issues. Any protocol amendments will be reviewed by all relevant parties including all site IRBs as necessary. Changes to the protocol that would alter the consent process will be communicated to study participants in an appropriate manor. The means for communicating any necessary changes to study participants will be determined by the research team taking into consideration the level of safety concern involved and with advice from the DSMB.

### Dissemination plans {31a}

Results of the study will be disseminated through meetings and publications. In disseminating results to study participants the research team will be consistent with the guidelines of the CTRI.

## Discussion

This study will assess the effectiveness of two formulations of single-dose IV iron in the treatment of IDA in pregnant women and reducing low birth weight deliveries when compared with oral iron, the current standard of care.

Though current research has demonstrated IV iron to be a safe and effective treatment of IDA in pregnant women, there remains a scarcity of large, quality trials that assess the effect on maternal and neonatal outcomes of iron administration in anaemic, pregnant women. The Cochrane Review indicates the need for assessing efficiency of iron treatment in pregnancy, specifically newer approaches such as single-dose IV iron. This study is unique in that it will address generalisability of this intervention through a more rigorous assessment on a large scale in a lower-middle income country. It also has the potential to influence the refinement of India’s health organizations current guidance on IDA treatment and lead to better long-term outcomes.

Another important aspect of this study is the independent economic analysis to be performed by experts in the field of health economics and cost-effectiveness of healthcare interventions. The study will report the cost-effectiveness when employing each single-dose IV iron formulation compared to oral therapy in the treatment of IDA in pregnant women. One aim is to demonstrate the economic feasibility of scale-up of this treatment approach and its subsequent usefulness as a treatment for IDA in pregnant women in other low- and middle-income countries with constrained resources. There is also an opportunity to analyse the economic impact of reducing low-birthweight and iron deficiency in the offspring on the cost of interventional programmes needed to modify the neurobehavioral effects of these two outcomes.

A potential challenge to consider is the impact that COVID-19 cases in India might have on recruitment and increased costs for personal protective equipment (PPE); however, every effort is being made to minimize the effects of these challenges.

While we have complete data for India’s National Family Health Survey (NFHS-4) for 2015–2016, new data for India’s NFHS-5 is currently being collected. The new data for Rajasthan is not yet available, however, the figures for Karnataka show an increase in the number of both children and pregnant women who are anaemic when compared to the 2015–2016 data [34]. This further underscores the need for a new, easily accessible and cost-effective approach for treating anaemia and timely importance of this study.

## Trial status

Protocol Version 1.2, March 8, 2021. Recruitment began on March 15, 2021. The projected completion date for recruitment is February 28, 2023.

## Abbreviations

ACOG: The American College of Obstetricians and Gynaecologists
AE: Adverse event
ASHA: Accredited Social Health Activist
CHC: Community healthcare centre
CIFF: Children’s Investment Fund Foundation
CMH: Cochran-Mantel-Haenszel
CRF: Case report form
CRO: Clinical research organization
CTRI: Clinical Trial Registry of India
DMS: Data management system
DSMB: Data Safety and Monitoring Board
DSMP: Data and Safety Monitoring Plan
EDD: Estimated delivery date
GCP: Good Clinical Practice
Hb: Haemoglobin
IDA: Iron deficiency anaemia
IRB: Institutional Review Board
ITT: Intent to treat
IV: Intravenous
JNMC: Jawaharlal Nehru Medical College
KAHER: KLE Academy of Higher Education and Research
LBW: Low birth weight
LMP: Last menstrual period
LRF: Laboratory Requisition Form
MOO: Manual of Operations
NFHS: National Family Health Survey
NICHD: National Institute of Child Health and Human Development
PHC: Primary healthcare centre
PI: Principal Investigator
PPE: Personal protective equipment
SAE: Serious adverse event
SMSMC: Sawai Man Singh Medical College
TAG: Technical Advisory Group
TJU: Thomas Jefferson University
TSAT: Serum transferrin saturation
WHO: World Health Organization
